# Five Different *Artemisia* L. Species Ethanol Extracts’ Phytochemical Composition and Their Antimicrobial and Nematocide Activity

**DOI:** 10.3390/ijms241814372

**Published:** 2023-09-21

**Authors:** Evgeny Nikitin, Igor Fitsev, Anastasia Egorova, Lidia Logvinenko, Dmitriy Terenzhev, Feruzakhon Bekmuratova, Adelya Rakhmaeva, Georgiy Shumatbaev, Alsu Gatiyatullina, Oksana Shevchuk, Tatiana Kalinnikova

**Affiliations:** 1 A.E. Arbuzov Institute of Organic and Physical Chemistry, Kazan Scientific Center, Russian Academy of Sciences, Arbuzov Str. 8, 420088 Kazan, Russia; berkutru@mail.ru (E.N.);; 2 A.M. Butlerov Chemical Institute, Kazan Federal University, Kremlevskaya Str. 18, 420008 Kazan, Russia; 3 Research Institute for Problems of Ecology and Mineral Wealth Use of Tatarstan Academy of Sciences, Daurskaya Str. 28, 420087 Kazan, Russia; 4 Nikitsky Botanic Gardens, National Scientific Center of Russian Academy of Sciences, 298648 Yalta, Russiaoksana_shevchuk1970@mail.ru (O.S.); 5 Federal State Budgetary Scientific Institution «Federal Center for Toxicological, Radiation, and Biological Safety», Nauchny Gorodok-2, 420075 Kazan, Russia; pantera08@yandex.ru

**Keywords:** *Artemisia annua* L., *Artemisia dracunculus* L., *Artemisia santonica* L., *Artemisia abrotanum* L., *Artemisia scoparia* Waldst. & Kit., ethanol extracts, chromatographic methods, mass spectrometry detection, antimicrobial activity, antifungal activity, nematocide activity, phytopathogens, nematodes, UNC-63 protein, nicotine receptor, *Caenorhabditis elegans*

## Abstract

Among the plants that exhibit significant or established pharmacological activity, the genus *Artemisia* L. deserves special attention. This genus comprises over 500 species belonging to the largest *Asteraceae* family. Our study aimed at providing a comprehensive evaluation of the phytochemical composition of the ethanol extracts of five different *Artemisia* L. species (collected from the southwest of the Russian Federation) and their antimicrobial and nematocide activity as follows: *A. annua* cv. Novichok., *A. dracunculus* cv. Smaragd, *A. santonica* cv. Citral, *A. abrotanum* cv. Euxin, and *A. scoparia* cv. Tavrida. The study of the ethanol extracts of the five different *Artemisia* L. species using the methods of gas chromatography–mass spectrometry (GC–MS) and high-performance liquid chromatography–quadrupole time-of-flight mass spectrometry (HPLC–MS/MS) allowed establishing their phytochemical profile. The obtained data on the of five different *Artemisia* L. species ethanol extracts’ phytochemical composition were used to predict the antibacterial and antifungal activity against phytopathogenic microorganisms and nematocidal activity against the free-living soil nematode *Caenorhabditis elegans*. The major compounds found in the composition of the *Artemisia* L. ethanol extracts were monoterpenes, sesquiterpenes, flavonoids, flavonoid glycosides, coumarins, and phenolic acids. The antibacterial and antifungal activity of the extracts began to manifest at a concentration of 150 µg/mL. The *A. dracunculus* cv. Smaragd extract had a selective effect against Gram-positive *R. iranicus* and *B. subtilis* bacteria, whereas the *A. scoparia* cv. Tavrida extract had a selective effect against Gram-negative *A. tumefaciens* and *X. arboricola* bacteria and *A. solani*, *R. solani* and *F. graminearum* fungi. The *A. annua* cv. Novichok, *A. dracunculus* cv. Smaragd, and *A. santonica* cv. Citral extracts in the concentration range of 31.3–1000 µg/mL caused the death of nematodes. It was established that *A. annua* cv. Novichok affects the UNC-63 protein, the molecular target of which is the nicotine receptor of the N-subtype.

## 1. Introduction

Today, biologically active compounds for preventing and avoiding plant diseases hold great promise for organic farming. It is well known that the chemically made pesticides conventionally used in the integrated agricultural complex are more dangerous for humans and the environment than substances of natural origin used in organic agriculture. The advancement of technological innovations in production and utilization, coupled with the increasing demand for organic goods, encourages the establishment of higher standards for human health and well-being, environmental safeguarding, and biodiversity, thereby enhancing overall food security [1].

At present, the quantity of formulations derived from natural compounds utilized for organic food production is significantly lower in comparison to the formulations employed in conventional agriculture. Therefore, it is essential to search for and develop new bio-based products for organic crop farming. One of the current trends is to utilize environmentally friendly plant extracts or essential oils as they are biodegradable, do not produce any toxic metabolites, and possess a wide range of antibacterial properties. This approach can effectively address the issue of pathogen resistance to plant protection chemicals.

Among the plants that exhibit significant or established pharmacological activity, the genus *Artemisia* L. deserves special attention. It includes more than 500 species belonging to the largest *Asteraceae* family [2,3]. This genus covers a diverse range of plants, which are widely distributed in the temperate climatic zones of Europe, Asia, North and South Africa, as well as North America. It comprises varieties of perennial, biennial, and annual herbs or small shrubs [4,5]. The literature review indicated that various species of *Artemisia* L. possess an unprecedented range of biological activities and are used in conventional folk and academic medicine [6]. The current biological activity studies of numerous *Artemisia* species do not reveal the full extent of the inherent formidable biochemical potential of this plant. Based on extensive empirical evidence, this plant was initially employed in antimalarial [7,8] and antibacterial therapy [9,10] and, subsequently, as antifungal [9,10,11,12,13] and antihepatotoxic [14,15] agents, with notable anti-spasmatic, anti-inflammatory, and angioprotective properties [6,16,17,18]. Tu Youyou, a Chinese pharmaceutical chemist, was awarded the Noble Prize in Physiology and Medicine [19,20] in 2015 for her efforts in extracting the artemisinin from *A. annua* L. [21] and synthesizing its derivatives, namely dihydroartemisinin [22], artemeter [23], and artesunate [24]. Additionally, she conducted a study on their pharmacological activity against *Plasmodium falciparum*, demonstrating the high potency of these substances against tropical malaria.

The plants of *Artemisia* are a valuable source of antioxidants [25], which serve as versatile stabilizers for membranes. Flavonoids, including flavones (apigenin, luteolin, jaceosidine, eryodictiol, etc.), flavonols (isorhamnetin), flavone glycosides (vitexin, luteolin-7-glucoside), and flavonol glycosides (kempferol 3-glucoside, rutin, kempferol 3-ramnoside, quercitrin, kempferol 3-rutinozid, and quercetin 3-galactoside) predominate in various species of *Artemisia* [26,27,28,29]. The most-common source of flavonoids, such as luteolin and eryodictiol, is *A. vulgaris* L. In addition to the conventional application of *Artemisia* species, there exist data demonstrating the cytotoxic properties of artemisinin against malignant cells [30,31]. Dehydroleukodin (sesquiterpen lactone) and dehydroparishin-B (sesquiterpen acid-B of the guayanic type), which also serve as active cytotoxicants of malignant cells, were extracted from the above-ground plants of *A. diffusa* and *A. douglassiana* [32,33].

Since various extracts of *Artemisia* species, as well as artemisinin and its derivatives demonstrate significant antivirulent activity [34,35], they were subsequently regarded as advanced remedies against HBV and HBC, herpes viruses HSV-1 and HSV-2, human immunodeficiency virus HIV-1, and influenza A virus [36,37,38,39] and also as a promising medicine against the new coronavirus infection, SARS-CoV-2, due to their powerful anti-inflammatory, immunoregulatory, and antiviral properties [40,41,42,43,44,45,46,47].

The available research data indicate that the biologically active potential of the various *Artemisia* species is far from being fully studied, and their conventional applications do not utilize their properties fully. Therefore, the current investigations [48,49,50,51,52,53,54,55] of the chemical compositions and biological activity of the various *Artemisia* L. species hold particular significance and are experiencing a real surge as they are presently in the focus of bioorganic and bioanalytical chemistry. The chromatography–mass spectrometry technique is widely acknowledged as the most-appropriate and -informative approach for the analysis of extracts or essential oils, specifically their chemical compositions, due to its ability to combine high separation power with reliable mass spectrometry detection [56]. However, the volatile compounds of plant extracts or essential oils with no confirmed thermal decomposition at gas transition temperatures should be separated using the method of gas chromatography–mass spectrometry (GC–MS) employing either electron or chemical ionization (EI or CI) techniques [57,58,59,60,61,62]. For polar and heavy components’ separation, it is advisable to use high-performance liquid chromatography (HPLC), which treats molecules leaving the chromatographic column with electrospray ionization (ESI) and, then, detects the generated particulates by a quadrupole time of flight mass spectrometer (HPLC–MS/MS-ESI) [63,64,65,66,67].

There are currently fourteen species of the *Artemisia* genus growing in the Aromatic and Medical Plants Collection of the Nikitsky Botanical Garden, having various ecological and geographical origins. Based on the introduction study, ten promising species were allocated for cultivated varieties, and five varieties were obtained for commercial cultivation in Crimea: *A. annua* cv. Novichok, *A. dracunculus* cv. Smaragd, *A. santonica* cv. Citral, *A. abrotanum* cv. Euxin, and *A. scoparia* cv. Tavrida.

The purpose of the present work was to study comprehensively the biochemical composition of five extracts of various *Artemisia* L. species selected by the Nikitsky Botanic Garden, specifically *A. annua* cv. Novichok, *A. dracunculus* cv. Smaragd, *A. santonica* cv. Citral, *A. abrotanum* cv. Euxin, and *A. scoparia* cv. Tavrida, utilizing the methods of GC–MS and HPLC–MS/MS-ESI, and to predict the antibacterial, fungicide, and nematocidal properties based on the identified chemical composition of these extracts.

## 2. Results and Discussion

### 2.1. Phytochemical Composition of Certain Species of Artemisia L. Extracts

Presently, a substantial quantity of information on the phytochemical properties has been accumulated for the plant extracts, resulting from the application of chromatography in conjunction with mass spectrometry detection and various methods of ionization [68,69,70]. The same applies to extracts and essential oils of various species of *Artemisia* L. The details of their phytochemical composition, derived from both qualitative and quantitative studies and given in [71,72,73,74,75], demonstrate the extensive array of bioactive compounds found in *Artemisia* L.

During the chromatographic analysis of five *Artemisia* L. species, the ethanol extracts were utilized in their native form without being diluted. The conditions of chromatographic fractionation were selected based on the literature data demonstrating that the extracts of various types of *Artemisia* L. have components that differ in their thermal stability, chromatographic lability, polarity, and boiling points. Hence, the examination of low-polar and volatile constituents of the prepared extracts was conducted by utilizing GC–MS with EI and CI. For the polar constituents, we utilized HPLC MS/MS (ESI+).

The results of GC-MC with different ionization types revealed a diverse range of major and minor constituents in the extracts of *A. annua* L., *A. dracunculus* L., *A. santonicum* L., *A. abrotanum* L., and *A. scoparia* L., as presented in Table 1. To identify the constituents, we used the relative match factor (RMF) generated by the NIST MS Search Program, on the automatic deconvolution of the mass spectra and their subsequent comparison with those in the reference digital libraries. The RMF of the compounds was largely within the range of 890–930 of the match factor, which proves the correct identification of the constituents found in the extracts. Only a third of the compounds had an RMF below 890. This is due to a weak peak intensity [M^+^] in the mass spectra with EI. In such cases, in order to ensure more-reliable identification [56,76], the peaks of higher intensity [M + H]^+^ were recorded by the CI method.

Analyzing the results of the study obtained by GC–MS (Table 1) for the extracts of *A. annua* L., *A. dracunculus* L., *A. santonicum* L., *A. abrotanum* L., and *A. scoparia* L. and the quantities of their constituents, we can conclude the following: they are composed of acyclic, monocyclic, and bicyclic monoterpenoids; monocyclic, bicyclic, and tricyclic and eudesmanic sesquiterpenoids; coumarins; phenolic compounds; and other chemical compounds. More detailed results are presented in the Table S1.

The acyclic monoterpenoids group revealed in five *Artemisia* extracts by the method of GC–MS are distributed as follows: β-myrcene, *trans*-β- and *cis*-β-ocimene, α-citral, and β-citral—in all tested extracts; Artemisia ketone—in *A. annua*, *A. santonica*, and *A. scoparia* extracts, with its highest content in *A. annua*; Artemisia alcohol—in *A. annua*, *A. dracunculus*, *A. santonica*, and *A. scoparia*. Furthermore, the acyclic diterpene alcohol of phytol was detected in the acyclic compounds of *A. annua*, *A. dracunculus*, *A. abrotanum*, and *A. scoparia*.

Monocyclic monoterpenoids such as limonene and cineole were found in *A. annua*, *A. dracunculus*, *A. santonica*, and *A. scoparia*. The group of bicyclic monoterpenoids was represented by 3-carene, α-pinene, β-pinene, and camphene found in all tested extracts; sabinene was found in *A. annua*, *A. dracunculus*, and *A. scoparia; cis*-sabinenehydrate—in *A. santonica* and *A. abrotanum;* camphor—in *A. annua*, *A. santonica*, and *A. abrotanum; cis*-chrysanthenol—in *A. santonica* and *A. abrotanum*; and borneol—in *A. annua*, *A. abrotanum*, and *A. scoparia*. Monocyclic sesquiterpenoids were represented by such compounds as α-curcumene (*A. santonica*, *A. scoparia*), germacrene D (*A. santonica*), and α-bisabolol (*A. annua*, *A. abrotanum*, *A. scoparia*). Bicyclic sesquiterpenoids: caryophyllene (*A. annua*, *A. santonica*, *A. abrotanum*, *A. scoparia*), α-eudesmol (*A. annua*, *A. santonica*, *A. abrotanum*), and arteannuic acid (*A. annua*, *A. dracunculus*, *A. scoparia*). Bicyclic sesquiterpene lactone (Arteannuin b) was found in *A. annua* solely. Tricyclic sesquiterpenoids were represented by such sesquiterpene alcohols as spathulenol (*A. annua*, *A. dracunculus*, *A. santonica*, *A. scoparia*) and isospathulenol (*A. dracunculus*, *A. santonica*, *A. abrotanum*). The group of coumarins detected by the GC–MS method was represented by four derivatives: 7-geranyloxycoumarin (*A. dracunculus*, *A. santonica*, *A. abrotanum*), scopoletin (*A. annua*), scoparone (*A. santonica*, *A. abrotanum*, *A. scoparia*), and isofraxidin (*A. annua*, *A. dracunculus*, *A. abrotanum*).

The phenolic compounds included estragole (*A. dracunculus*, *A. santonica*, *A. abrotanum*), with its highest content in *A. dracunculus*, eugenol (*A. santonica*, *A. scoparia*), and capillene (*A. dracunculus*, *A. santonica*, *A. abrotanum*, *A. scoparia*).

In addition to the compounds listed above, using the GC–MS method in the extracts of *A. annua*., *A. dracunculus*, *A. santonica*, *A. abrotnum*, and *A. scparia*, the following were detected: sesquiterpene hydrocarbon (*E*)-β-famesene (*A. annua*, *A. santonica*, *A. abrotanum*); eudesmane sesquiterpenoid β-selinene (*A. annua*, *A. santonica*); alkamides (pellitorine, (2*E*,4*E*)-*N*-isobutylundeca-2,4-dien-8,10-diynamide) (*A. annua*, *A. dracunculus*, *A santonica*, *A. abrotanum*); (2*E*,4*E*)-1-(piperidin-1-yl)deca-2,4-dien-1-one (*A. dracunculus*); a capillin (*A. dracunculus*, *A. santonica*). The last one can be considered as the structure contains acetophenone and a polyyne (pentadiynyl) portion, conjugated together as an ynone. *A. scoparia* was detected to contain α-Amyrin, a pentacyclic triterpenoid alcohol.

The compounds detected in each *Artemisia* L. extract are enlisted in Table 2 with their names, molecular formula, experimental masses, ppm error < 5 (Table S2), and retention time values. Based on the results obtained by the HPLC–MS/MS(ESI) technique for *A. annua*, *A. dracunculus*, *A. santonica*, *A. abrotanum*, and *A. scoparia* and the quantitative analysis of the identified components (Table 2), it can be concluded that these species comprised sesquiterpene lactones such as β-santonin, artemisinin (*A. dracunculus*, *A. santonica*, *A. abrotanum*, and *A. scoparia*), taurin (*A. annua*, *A. dracunculus*, *A. santonica*, and *A. scoparia*), bicyclic sesquiterpenoid—arteannuic acid—(*A. annua*, *A. dracunculus*, and *A. scoparia*), and bicyclic sesquiterpene—arteannuin b (*A. annua*). The group of phenolic acids consisted of gentisic acid (*A. annua* and *A. scoparia*), chlorogenic acid, 3-*O*-feruloylquinic acid, ferulic acid, isochlorogenic acid, and rosmarinic acid detected in each of the five *Artemisia* L. extracts. During the HPLC analysis, we obtained records of peaks and mass spectra (ESI+) of coumarins including scopoletin (*A. annua* L.), which is consistent with the results obtained by the GC–MS, and of the coumarin glucosides, i.e., scopolin (*A. annua* L.), isofraxidin (*A. annua*, *A. dracunculus*, *A. abrotanum*), and fraxidin, identified in each of the five Artemisia extracts. The most-numerous groups identified by HPLC–MS/MS were composed of the following flavonoids: luteolin, isorhamnetin, eupatolitin, chrysosplenol D, and cirsilineol (*A. annua*, *A. dracunculus*, *A. santonica*, *A. abrotanum*, *A. scoparia*), quercetin-3-*O*-hexoside (*A. abrotanum*, *A. scoparia*), and casticin (from plant extracts of *A. santonica* and *A. scoparia*); flavonoid glycosides (rutin, quercetrin, and isoquercetrin) identified in every studied species of *Artemisia*; and flavonoid glucosides, namely vicenin-2 (*A. annua*, *A. dracunculus*, *A. abrotanum*, *A. scoparia*), feruloyl glucose (*A. annua*, *A. dracunculus*, *A. santonica*), and luteolin 7-*O*-glucoside (*A. santonica*, *A. abrotanum*, *A. scoparia*). Apart from that, *A. abrotanum* and *A. scoparia* were found to contain isorhamnetin-3-rutinoside, which belongs to the estrogenic flavonoids [77], similar to luteolin, isorhamnetin, rutin, quercetrin, isoquercetrin, and vicenin-2.

Extracted by the GC–MS and HPLC–MS/MS techniques, the collection of chemical compounds constituted a distinct and unique phytochemical profile composed by the five different species (*A. annua* cv. Novichok, *A. dracunculus* cv. Smaragd, *A. santonica* cv. Citral, *A. abrotanum* cv. Euxin, *A. scoparia* cv. Tavrida), exhibiting high biological activity and exercising antimicrobial properties attributed to terpenoids and sesquiterpenoids. Furthermore, β-santonin, a constituent of the sesquiterpene lactones group, has a wide spectrum of activity against nematodes and cestodes.

### 2.2. Antibacterial and Antifungal Activity of Ethanol Extracts of Artemisia L. Plants

Based on the literature data, the *Artemisia* L. essential oils and plant extracts possess high antibacterial activity against a wide spectrum of pathogenic microorganisms.

Thus, the *Artemisia* L. essential oils effectively inhibit the growth of the following fungal species: *Rhizoctonia solani*, *Sclerotium rolfsii*, *Macrophomina phaseolina*, *Colletotrichum fragariae*, *Colletotrichum gloeosporioides*, *Colletotrichum acutatum*, etc., which cause diseases and high crop losses [25,78,79,80]. The *A. scoparia* L., *A. sieberi* L., and *A. aucheri* L. essential oils and extracts were found to be effective against phytopathogens [71,81,82]. The antibacterial properties are largely dependent on the time of year when the plants are harvested to produce extracts, essential oils, and BAC; the highest potential was demonstrated by plants prepared in the second half of summer [83].

In a series of laboratory experiments with serial dilution, the antibacterial effectiveness of the *Artemisia* L. ethanol extracts against the phytopathogenic bacteria (four strains: *Rathayibacter iranicus* and *Bacillus subtilis* (Gram-positive) and *Agrobacterium tumefaciens* and *Xanthomonas arboricola* (Gram-negative) and phytopathogenic fungi (three strains: *Alternaria solani*, *Fusarium graminearium*, and *Rhizoctonia solani*) was evaluated for the first time. The results of the studies are given in Table 3 and Table S3.

The antibacterial properties of the extracts varied depending on the species of the plant and pathogenic strain. The extract from *A. annua* was the most-effective against the Gram-positive bacteria of *R. iranicus*, with the minimal inhibitory concentration (MIC) being 500 µg/mL and the minimal bactericidal concentration (MBC) being 1000 µg/mL. The antibacterial activity of the *A. annua* extract was likely mediated by the presence of artemisinic acid, ketone, and alcohol in the Artemisinin extract and its precursors [84]. Another Gram-positive *B. subtilis* strain was more resistant to the components of the *A. annua* extract. For *B. subtilis*, the inhibitory concentration was within 2000 µg/mL and above.

The ethanol extract of *A. annua* demonstrated low antibacterial and antimycotic properties against the studied Gram-negative phytopathogenic bacterial and fungal strains. The concentrations inhibiting the growth of *X. arboricola* and *R. solani* were, respectively, 1000 µg/mL and 2000 µg/mL, while 4000 µg/mL and higher for *A. tumefaciens*, *A. solani*, and *F. graminearum*. The antimicrobial properties of the *A. annua* extract prepared from cv. Novichok (the Nikitsky Botanic Garden) were comparable to those of the *A. annua* methanol extracts from India, but they were lower than those of the *A. annua* methanol extracts from Bulgaria [85,86] and significantly higher than those of the ethanol extracts from the plants grown in Romania [87]. Among all the species under study, the *A. dracunculus* ethanol extract demonstrated a selective antimicrobial activity against *R. iranicus*, requiring the lowest inhibitory and bactericidal concentrations. At an extract concentration of 310 µg/mL, the bacterial cells of R. *iranicus* were killed. According to the *Alvarez-Martínez* and *Van Vuuren* method, which distinguishes plant extracts by their effectiveness against bacteria and fungi, the obtained results correspond to a very high antimicrobial effect [88,89]. Such data are likely to be associated with the high estragole content attributed to the extracts of these species, which demonstrates large areas of inhibited Gram-positive bacteria growth [90]. Furthermore, estragole is famous for its antimycotic effect against pathogens as such *A. niger*, *A. flavus*, *T. viride*, *C. albicans*, *A. solani*, *F. graminearum*, and *R. solani* [91,92]. As per our research findings, the effective concentration of the *A. dracunculus* ethanol extract for inhibiting fungal growth is 1120 µg/mL. The obtained findings were similar to the results for the *A. santonica* ethanol extract.

*X. arboricola*, a Gram-negative bacterium, was more sensitive to the *A. dracunculus* and *A. santonica* extracts compared to *B. subtilis* and *A. tumefaciens*, and it ceased growing when the extracts’ concentrations reached 625 and 560 µg/mL, respectively.

The antimicrobial activity of the *A. dracunculus* ethanol extract was lower than that of the aqueous extract and higher than that of the methanol extract and was comparable to that of the *A. tarragona* ethanol–water extract from Iran, with the exception of the antimicrobial activity against *R. iranicus* and Gram-negative bacteria. Our experiments had significantly lower results for minimal inhibitory concentrations [82,93,94].

The extract of *A. santonica* demonstrated significantly higher outcomes compared to those obtained for the anti-phytopathogenic extracts [95] and lower than those obtained for the methanol extracts prepared from Bulgarian plants [86].

The ethanol extract of *A. scoparia* in comparison to others demonstrated significant effectiveness against Gram-negative bacteria (*X. Arboricola* and *A. Tumefaciens*) and fungi (*F. graminearum* and *R. solani*). Thus, the MIC values for *X. arboricola* and *R. solani* were 150 µg/mL, *F. graminearum*—310 µg/mL, and *A. tumefaciens—*625 µg/mL. These values correspond to the very high antimicrobial activity of the extracts [88,89]. The minimum bactericide and fungicide concentrations of the *A. scoparia* L. extract for the *X. arboricola*, *R. solani*, and *F. graminearium* strains were 625 µg/mL, being 2–4-times higher than the MIC values for the same. The values of the antibacterial activity of the ethanol extract of *A. scoparia* significantly exceed the activity of plants grown in Iran and Palestine [80,96].

The prominent antimicrobial effect of the *A. scoparia* ethanol extract is probably attributable to the high contents of capillin, capillen, and scoparon. The percentage of their total presence in the extract was 63%. Scoparon is a phytoalectin, a secondary metabolite produced by plants as a reaction to contamination by various pathogens [97]. Capillen and capillin are the major components of the essential oils, manifesting their strong antibacterial and antimycotic properties against various pathogens that cause diseases in animals and humans [98,99,100,101].

The extract of *A. abrotanum* cv. Euxin demonstrated the lowest activity among the studied ethanol extracts. Its MIC, MBC, and MFC values were 2000 µg/mL and higher. The concentrations inhibiting the fungal growth and killing their cells have not been determined for fungal species. According to Alzoreky N.S. [102], the antibacterial concentrations of the *A. abrotanum* methanol extract from Yemen started from 1320 µg/mL, which is in line with the data obtained in our studies.

Therefore, as per our study, the following antimicrobial sequence can be stated for the ethanol extracts of the *Artemisia* L. genus plants: *A. scoparia* > *A. dracunculus* > *A. santonica* > *A. annua* > *A. abrotanum*.

### 2.3. Nematocidal Activity of cv. Artemisia L. Extracts

*Caenorhabditis elegans*, a free-living soil nematode, is a suitable model organism for studies in neurotoxicology and ecotoxicology. *C. elegans* has an advantage over other model organisms due to its easy and inexpensive cultivation in the laboratory, safety for scientists, and high fertility [103,104,105]. *C. elegans* consists of 959 somatic cells including 302 neurons [104]. The nervous system of *C. elegans* has 890 electrical synapses, 1410 neuromuscular synapses, and 6393 chemical synapses using neurotransmitters specific to the vertebrate body (acetylcholine, dopamine, serotonin, glutamate, and γ-aminobutyric acid) [103]. At the same time, nematodes lack a circulatory system and an organ of external respiration, thereby significantly facilitating the interpretation of the results of toxicological experiments [104]. Ethanol extracts of various raw materials *A. annua*, *A. dracunculus*, and *A. santonica*, were found to cause dose-dependent nematode mortality (Table 4).

(−)-Tetramisole hydrochloride was used as a comparator. At concentrations of 1.875 μg/mL, 3.75 μg/mL, and 7.5 μg/mL, tetramisole caused the death of 14.5 ± 2.5, 26.0 ± 3.1, and 61.5 ± 2.7% of nematodes in 24 h.

In order to identify potential molecular targets of the studied extracts’ action, we performed experiments to evaluate their toxicity at a concentration of 500 µg/mL for *C. elegans* of both the wild-type and mutant lines.

Table 5 provides the results of the experiments aimed at examining the effects of the Artemisia extracts on nematodes of the N2 wild-type and the mutant lines of RB896, RB756, and JD217 with null mutations in the genes encoding the muscarin receptors of acetylcholine such as gar-1, gar-2, and gar-3, respectively. The RB918 line is a null mutation of the nicotine receptor gene for N-subtype acetylcholine. The *A. annua* extract for *C. elegans* of the RB896, RB756, and RB918 lines was the least toxic extract in these experiments. Three subtypes of the above receptors have been found in *C. elegans*, GAR-1, GAR-2, and GEAR-3, respectively [106,107,108]. The GAR-3 receptor is the best-investigated one; as for the pharmacological profiles of the GAR-1 and GAR-2 receptors, they are understudied. The low toxicity of the *A. annua* extract for nematodes of the RB896 and RB756 lines indicates that this extract contains substances affecting the GAR-1 and GAR-2 mAChRs of *C. elegans* (Table 5).

The agonists of the acetylcholine nicotine receptor (nAChRs) are represented by a large group of substances and used as pesticides and anthelminthic drugs. Nicotine receptors of both vertebrates and invertebrates are the ion channels formed by five transmembrane proteins. Homomeric and heteromeric agonists of acetylcholine nicotine receptors (nAChRs) are distinguished. The N-type nicotine receptor in *C. elegans* is homomeric and consists of five identical protein subunits of ACR-16 [109,110]. The results of our experiments showed that the extract of *A. annua* contains substances whose molecular target is the N-type nicotine receptor (Table 6).

Table 6 shows the toxicity of the artemisia extracts for the nematode line of the N2 wild-type and the nematodes of the five mutant lines. Each line lacks one of five subunits of the L-type nicotine receptor sensitive to levamisole. In most cases, the sensitivity of the *C. elegans* mutant lines to the negative effects of the Artemisia extracts did not exceed the sensitivity of the N2 wild-type line. The exception was the effect of the *A. santonica* extract on the CB904 nematode line and the effect of the *A. annua* extract on the CB522 nematode line. The least-toxic extract was *A. annua* for *C. elegans* of the VC428 line with the null mutation of the UNC-63 gene. The low sensitivity of *C. elegans* of the VC428 line to the toxic effect of the extract of *A. annua* L. suggests that this extract contains substances whose target is one of the subunits of the nAChR L-subtype, namely the UNC-63 protein.

Now, the study of nicotine receptors in invertebrates has a strong focus as these receptors are targets of many pesticides and anthelminthics. All nicotine acetylcholine receptors consist of five protein subunits, but there are more genes encoding these receptors. *C. elegans* has 29 genes of such a type, human—17—and investigated invertebrates—from 7 to 12 [110]. Hence, a large number of subtypes of these receptors with selective sensitivity to toxicants can be expected. Levamisole-sensitive L-subtype nicotine receptors are present in nematodes, both free-living and parasitic, but they are missing in warm-blooded animals, allowing the use of these receptor agonists for the treatment of helminthiasis in humans and animals. *C. elegans* has an L-subtype nicotine receptor consisting of three α-subunits (LEV-8, UNC-38, and UNC-63) and three non-α-subunits (UNC-1 and UNC-29). The α-subunits are thought to be involved in the binding of acetylcholine and other N-cholinergic agonists, and the other subunits form the ion channels in neuronal membranes and locomotor muscles [110,111,112]. Our mutant analysis revealed that the *A. annua* extract has substances affecting the nAChR N-subtype, consisting of five molecules of the ACR-16 protein (Table 4), and also, the α-subunit of the UNC-63 receptor is affected by the substances derived from the *A. annua* extract (Table 6).

## 3. Materials and Methods

### 3.1. Sampling

For the raw material, we used *A. annua* L. (Novichok variety), *A. dracunculus* L. (Smaragd variety), *A. santonicum* L. (Citral variety), *A. abrotanum* L. (Euxin variety), and *A. scoparia* Waldst. & Kit (Tavrida variety) from an experimental area of the Aromatic and Medical Plants Collection in the Nikitsky Botanical Garden, located on the southern coast of Crimea (44°31′ N, 34°15′ E, 200 m above sea level). The plants grew in brown carbonate soil with medium humus and low clay content. The climatic conditions of the southern coast of Crimea are attributed to the geographical features, specifically the mountains, which shield the coastline from the northern winds and the warm waters of the Black Sea. All these have resulted in a dry subtropical climate of a Mediterranean type.

The sampling for the study was conducted during the mass flowering of the plants. The raw material was collected between 11 a.m. and 2 p.m., during the hottest hours of the day. The portion above ground was trimmed at the level of the lower leaves of the plant. The collected plants were stored in a well-ventilated location, shielded from direct sunlight, at a temperature range of 30–35 °C.

### 3.2. Extracts’ Preparation

A single-stage maceration was used to prepare five types of extracts from *A. annua*, *A. dracunculus*, *A. santonica*, *A. abrotanum*, and *A. scoparia*. The dry plant material was ground to a particle size range of 1–2 mm in a LM 202 laboratory mill manufactured by NV-Lab (Moscow, Russia). The ground material test portions, each weighing 15.0 g, were poured with 150 mL of a 70% ethanol solution and macerated at 45 °C under constant stirring for an hour and a half. The prepared extracts were filtered using a first-type Whatman filter (Cat. No. 1001 110). The obtained filtrate materials were concentrated in a LabTex Re 100-Pro rotary evaporator manufactured by DLAB Scientific Co., Ltd., Beijing, China, and stored in a dark location at a temperature of 4 °C.

### 3.3. Gas Chromatography–Mass Spectrometry

In the context of GC–MS studies, the recording of chromatograms was carried out for a full-ion current by utilizing a Chromatec Crystal 5000.2 gas chromatograph, which was paired with Chromatec, a quadrupole mass spectrometer detector equipped with an Advanced Ion Source (Chromatec, Yoshkar-Ola, Russia), for both electron ionization (EI) and chemical ionization (CI). CR–5MS, a quartz capillary column (5%-phenyl-, 95%-dimethyl polysiloxane, 30 m × 0.25 mm × 0.25 µm, Chromatec, Yoshkar-Ola, Russia), was utilized. The GC–MS test conditions were as follows: injector temperature of 280 °C; interface temperature of 270 °C; ion source temperature of 250 °C; column oven initial temperature of 60 °C; initial thermostat conditioning time of 1 min; column temperature rate of 5°/min up to 210 °C; subsequent temperature rate of 12°/min up to 280 °C; isothermal conditions for 40 min; gas-carrier (He, 99.999%) velocity of 0.9 mL/min; sample volume of 1 µL; split ratio of 1:100.

The *Artemisia* L. extract composition was determined by recording the mass spectra for EI and CI at 70 eV and 30 eV, respectively, within the range of 50–550 *m*/*z* (using CH_4_ reagent gas, high purity). In order to process the mass spectra, we used the Chromatec Analytic 3.5 software (Chromatec, Yoshkar-Ola, Russia), the NIST MS Search Program, V.2.3, the NIST’20 mass spectra electronic libraries (NIST, Gaithersburg, MD, USA), and Wiley 12th Edition (Wiley, Hoboken, NJ, USA) [113].

In addition, the retention times and indices were compared with those reported in [114,115] and presented in the databases mentioned above.

### 3.4. High Performance Liquid Chromatography—Mass Spectrometry

The HPLC–MS/MS-ESI study was performed on a Bruker maXis II™ high-resolution ESI-QTOF mass spectrometer (Bruker GmbH, Bremen, Germany) equipped with the ESI source Apollo II (in positive-ion-detection mode, capillary voltage +4 KV, source temperature of 250 °C, voltage shift of 500 V at the end plate, scanning time of 0.2 s) and coupled to the HPLC Dionex UltiMate™ 3000 series (Thermo Fisher Scientific, Waltham, MA, USA), including a gradient pump, a degassing module, an automatic sampler, and a chromatographic oven. The SB-C18 Agilent Zorbax chromatographic column (150 mm × 4.6 mm, 5 µm, Agilent Technologies, Santa Clara, CA, USA) was applied with the guard column (25 mm × 4.6 mm, 5 μm, Agilent Technologies, Santa Clara, CA, USA).

HPLC–MS/MS-ESI conditions: a binary eluent (Solvent A: 0.1% aqueous formic acid; Solvent B: acetonitrile). The gradient elution mode: 5% of B for 0–22 min, 5–15% of B for 22–35 min, 15% of B for 35–45 min, 15–20% of B for 45–55 min, 20–50% of B for 55–60 min, 50–65% of B for 60–80 min, 65% of B for 80–85 min, and 5% B for 85–90 min at a constant flow rate of 0.6 cm^3^/min, 30 °C column oven, and injection volume of 10 µL.

Mass spectra (ESI+) were recorded within the range of 100–1000 Da. Auto MS/MS, the automatic fragmentation mode, was applied, and the collision energy parameters were set as follows: 15 eV (within the range of 100–200 Da), 35 eV (200–500 Da), 50 eV (500–1000 Da).

N_2_ was used (high purity) as the drying (flow rate of 8 L/min at a temperature of 250 °C), spray (pressure of 4 bar), and collision gas.

The Compass Data Analysis 4.4, Compass Target Analysis 4.4 (Bruker GmbH, Bremen, Germany) software was used to synchronize the work with the HPLC Dionex UltiMate™ 3000 series and control the mass spectral data collection and handling.

Using target analysis, all compounds were detected by applying strict analyzing criteria. Different collision energies were applied to obtain good-intensity parent ions, as well as significant fragment ions. Accurate and reproducible spectra with high mass accuracy helped in the identification process. The acquired data were processed using three acquisition software including Compass Data Analysis 4.4, Compass Target Analysis and Compass Library Editor (Bruker GmbH, Bremen, Germany). The MS intensity threshold was set at >1000, MS^2^ intensity > 500, base peak intensity 1000 counts (minimum), ppm error > 5, and mSigma values > 50.

GC–MS and HPLC–MS/MS were run in three replications. Statistical treatment of the data obtained was performed at a significance level of 5%. The results are presented as the average value ± the coverage interval.

Correlation analysis was performed with the OriginPro 8.1 software (OriginLab Corp., Northampton, MA, USA).

### 3.5. Reagents

The reagents used were as follows: deionized water (resistivity at 25 °C equal to 18 MΩ·cm) obtained with the Barnstead Pacific-TII Unit (Thermo Fisher Scientific, Waltham, MA, USA), acetonitrile (gradient for chromatography), formic acid (98–100% “chemically pure pharm.”, Merck, Darmstadt, Germany), ethyl alcohol (“high purity”), norfloxacin, (−)-tetramisole scopoletin, rutin, luteolin, and artemisinin, (purities ≥98%) from Sigma-Aldrich Co., (Sigma-Aldrich Co., St. Louis, MO, USA), chloramphenicol (Kazan Pharmaceutical Plant, Kazan, Russia), and difenoconazole (Score250 EC, Syngenta, Wilmington, DE, USA). In ethanol, stock standard solutions (1 mg/mL) of scopoletin, rutin, luteolin, and artemisinin were produced and kept at 4 °C.

### 3.6. Antibacterial Effect Tests

#### 3.6.1. Microorganism Strains and Growth Media

The study utilized the following phytopathological strains, namely bacteria (*Rathayibacter iranicus* ECM Ac-1602, *Bacillus subtilis* ECM B-12, *Xanthomonas arboricola* S3, *Agrobacterium tumefaciens* A-47) and fungal species (*Alternaria solani* K-100054, *Fusarium graminearum* FG-30, *Rhizoctonia solani* BKM F-895).

The liquid broth containing the spores of the microorganisms was prepared using standard growth media: Potato Extract Glucose broth for *B. subtilis* EMC B-12, *X. arboricola* S3, *A. Tumefaciens* A-47, *A. solani* K-100054, *F. graminearum* FG-30, and *R. solani* EMC F-895 and Corynebacterium Selective Agar for *Rathayibacter iranicus* EMC Ac-1602.

#### 3.6.2. Antimicrobial Assay In Vitro

In the experiments, the determination of the minimum inhibitory concentration (MIC) of the extracts of the following cultivar plants, *A. annua*, *A. dracunculus*, *A. santonica*, *A. abrotanum*, and *A. scoparia*, was performed using the method of two-fold serial dilution [116] with the modification provided in [117,118]. Fungistatic activity was defined by the serial dilution method [119] in liquid medium.

The 24 h bacterial cultures a swell as the fungal cultures grown from the 7th to the 14th d were used in the study. The final inoculum contained 10^5^ CFU/mL of bacteria and 1.1–1.5 × 10^2^ CFU/mL of fungi. The bacteria concentration was determined using a DEN-1B densitometer (Biosan, Riga, Latvia). Tubes containing only culture medium were used as the control.

In order to establish the minimal concentrations of the bactericidal and fungicidal agents (MBC and MFC, respectively), 10 mL of inoculum (or a piece of fungal mycelium) obtained from tubes with no discernible growth was incorporated into Petri dishes containing agarized nutrient medium via an inoculation loop.

The results of the bacterial growth were observed every day for 5 days at 30 °C for *R. iranicus* VKM Ac-1602, *B. subtilis* VKM B-12, and *A. tumefaciens* A-47 and at 25 °C for *X. arboricola* S3. The fungus was incubated in a thermostat at 26 °C for 7 d. The growth of the microorganisms was visually determined [76].

Norfloxacin, chloramphenicol, and difenoconazole were used as reference compounds in the experiments.

### 3.7. Nematocidal Activity

The following lines of *Caenorhabditis elegans* received from Caenorhabditis Genetics Center were used in this work: the N2 wild-type line and mutant lines of CB211 (*lev-1(e211)*IV), CB522 (*unc-29(e522)*I), CD904 (*unc-38(e264)*I), JD217 (*gar-3(vu78)*V), RB756 (*gar-2(ok520)*III), RB896 (*gar-1(ok755)*), RB918 (*acr-16(ok789)*V), VC428 (*unc-63(gk234)*I), and VC1041 (*lev-8(ok1519)*X). *C. elegans* was cultured in Petri dishes (d = 10 cm) with standard nematode-growing medium, which consisted of 17 g/L of bactoagar, 2.5 g/L of peptone, 50 mM NaCl, 1 mM MgSO_4_, 1 mM CaCl_2_, 5 mg/L of cholesterol, and 25 mM potassium phosphate buffer (pH 6.0). *E. coli* strain OP50 was used for nematode feeding [116]. Experiments were carried out with young mature nematodes (young adults) incubated in M9 buffer (3 g/L KH_2_PO_4_, 6 g/L Na_2_HPO_4_, 5 g/L NaCl, 1 mM MgSO_4_). The nematodes were washed three times for each experiment to remove the culture medium, bacteria, and exometabolites in M9 buffer with a volume of 10 mL and 50 individuals placed per vial in glass centrifugal vials of 10 mL. The M9 buffer and extracts of the *A. annua*, *A. dracunculus*, and *A. santonica* plants were added to the vials. To determine the nematocidal activity, the extracts were redissolved in DMSO. The final incubating volume was 1 mL. The number of dead nematodes was counted in 24 h. The nematodes that were incapable of responding to mechanical stimulus were deemed dead.

All studies were conducted with a three-fold repetition, and the results of the definitions were handled in accordance with the international standards [120,121].

## 4. Conclusions

The ethanol extracts of *Artemisia* L. cultivated on the southern coast of the Crimean peninsula in the Nikitsky Botanical Garden contain acyclic, monocyclic, and bicyclic monoterpenoids, monocyclic, bicyclic, tricyclic, and eudesmane sesquiterpenoids, coumarins, and phenolic compounds as the main compounds, having antibacterial and antifungal activity against phytopathogenic microorganisms.

The ethanol extract of the *A. scoparia* of Tavrida variety was the most toxic for microscopic phytopathogenic fungi and Gram-negative bacteria, and the *A. dracunculus* extract of the Smaragd variety had a toxic effect on Gram-positive bacteria. The minimum inhibitory concentrations of the above extracts were 150 µg/mL due to their high antimicrobial activity.

The nematocidal activity of the ethanol extracts of *A. annua* of the Novichok variety, *A. dracunculus* of the Smaragd variety, and *A. santonica* of the Citral on *C. elegans*, a soil nematode, characterized by a concentration range of 31.3–1000 µg/mL, was established. The mutant analysis revealed the insignificant effects of *A. dracunculus* and *A. santonica* extracts on the muscarinic and nicotinic receptors of *C. elegans*. Moreover, the *A. annua* extract had compounds acting on both the N-subtype nAChR receptors and levamisole receptors of α-subunit UNC-63.

The total results of the studies on the ethanol extracts obtained from the five varieties of the *Artemisia* L. species growing in the Nikitsky Botanical Garden collection of aromatic and medical plants indicated that, due to their high content of natural chemical compounds of different classes, showing high antibacterial, antifungal, and nematocidal activity, they are advanced initial raw materials as a basis for designing in the future the most-advanced and selective-goods for organic agriculture using the natural potential of the plants (Tables S1–S3).

## Figures and Tables

**Table 1:** The results of the determination of the quantitative content of non-polar components in extracts of the plants as follows: *A. annua*. cv. Novichok, *A. dracunculus* cv. Smaragd, *A. santonica* cv. Citral, *A. abrotanum* cv. Euxin, and *A. scoparia* cv. Tavrida obtained by GC–MS (components with ω > 0.04%, mass., *n* = 3, *p =* 0.95).

*t_R_*^1^, min	Compound	ω (%)				
		*A. annua* cv. Novichok	*A. dracunculus* cv. Smaragd	*A. santonica* cv. Citral	*A. abrotanum* cv. Euxin	*A. scoparia* cv. Tavrida
1	3	6	7	8	9	10
6.38	α-pinene	0.52	0.21	7.94	0.083	5.24
6.43	Camphene	3.34	1.10	2.99	8.10	0.09
6.66	β-pinene	0.84	1.01	0.54	0.09	5.68
6.96	Sabinene	1.26	0.06	–	–	1.41
7.08	β-myrcene	9.82	1.35	8.02	0.06	0.54
7.11	3-carene	2.16	1.30	0.09	0.05	0.04
7.83	Limonene	1.87	3.61	3.54	–	2.80
7.90	*cis*-β-ocimene	1.0	1.37	1.23	–	–
7.78	Zineol	11.76	4.23	1.56	–	0.11
7.99	*trans*-β-ocimene	1.02	0.05	14.51	23.21	0.09
8.17	Artemisia ketone	32.81	–	1.21	–	0.05
8.53	Artemisia alcohol	3.74	0.06	0.05	–	0.08
8.94	*cis*-sabinenehydrate	–	–	1.58	0.08	–
9.71	Camphor	10.10	–	1.32	1.29	–
9.86	*cis*-chrysanthenol	–	–	21.57	34.26	–
10.07	Borneol	1.95	–	–	1.43	2.05
10.37	Estragole	–	56.20	4.86	3.34	–
10.90	α-citral	0.63	1.28	2.85	0.35	1.83
11.30	β-citral	1.02	0.91	3.74	0.38	2.02
12.53	Eugenol	–	–	4.73	–	1.70
13.63	Caryophyllene	0.73	–	0.04	0.05	3.02
13.79	(*E*)-β-famesene	1.96	–	1.05	0.19	–
14.23	α-curcumene	–	–	0.13	–	2.34
14.39	Germacrene D	–	–	0.07	–	–
14.41	β-selinene	1.70	–	3.29	–	–
14.50	Capillene	–	0.04	1.23	3.54	44.61
15.54	Capillin	–	0.05	0.87	–	7.46
16.07	Spathulenol	0.16	2.89	0.82	–	3.83
16.15	Isospathulenol	–	1.60	5.48	0.06	–
16.45	α-eudesmol	0.08	–	0.17	1.43	–
16.63	α-bisabolol	0.40	–	–	8.18	0.52
17.12	Arteannuin b	4.00	–	–	–	–
18.22	Arteannuic acid	4.05	1.24	–	–	0.86
19.09	(2*E*,4*E*)-*N*-isobutyl-2,4-undecadiene-8,10-diynamide	0.06	2.61	0.15	0.54	–
19.46	Pellitorine	–	3.64	1.97	0.93	–
19.52	Scopoletin	2.08	–	–	–	–
20.58	Scoparone	–	–	0.26	2.01	10.93
20.60	Isofraxidin	0.08	0.24	–	0.10	–
20.75	Phytol	0.81	1.68	–	10.01	0.98
21.02	(2*E*,4*E*)-1-(piperidin-1-yl)deca-2,4-dien-1-one	–	3.19	–	–	–
21.14	7-geranyloxycoumarin	–	10.05	2.25	0.20	–
29.76	α-Amyrin	–	–	–	–	1.70

^1^ Retention time, min.

**Table 2:** The results of quantitative content determination for polar components in the extracts of *A. annua* cv. Novichok, *A. dracunculus* cv. Smaragd, *A. santonica* cv. Citral, *A. abrotanum* cv. Euxin, and *A. scoparia* cv. Tavrida obtained by the HPLC–MS/MS(ESI+) technique (the components with ω ≥ 0.04%, in mean ± SEM, n = 3, *p* = 0.95, are provided).

*t_R_*^1^, min	Compound	ω (%)				
		*A. annua * cv. Novichok	*A. dracunculus * cv. Smaragd	*A. santonica* cv. Citral	*A. abrotanum* cv. Euxin	*A. scoparia* cv. Tavrida
1	2	8	9	10	11	12
2.4	Arteannuic acid	4.85	1.74	–	–	0.92
15.0	Gentisic acid	0.57	0.52	0.48	0.10	0.58
33.5	Scopolin	2.09	–	–	–	–
35.8	Isofraxidin	1.11	2.28	2.54	2.22	2.12
36.7	Feruloyl glucose	1.71	4.92	1.53	4.63	4.23
38.5	Vicenin-2	0.06	4.61	–	2.04	6.08
40.8	3-*O*-feruloyl-quinic acid	3.84	4.61	2.73	4.52	4.32
46.8	Scopoletin ^2^	2.06	–	–	–	–
49.9	Fraxidin	1.04	2.05	2.05	2.02	1.61
51.3	Rutin ^2^	3.23	2.28	4.14	5.04	2.98
52.3	Isoquercetin	3.39	3.91	4.25	3.35	0.63
53.5	Quercetin-3-*O*-hexoside	4.73	2.94	4.83	4.83	5.33
53.8	Luteolin 7-*O*-glucoside	3.43	3.32	3.34	2.78	7.24
57.2	Isochlorogenic acid	3.04	3.45	3.56	3.22	6.34
58.5	Isorhamnetin-3-rutinoside	4.44	3.35	3.23	4.22	–
58.9	Quercetrin	6.41	6.74	7.69	6.53	2.10
61.4	Luteolin ^2^	3.32	3.57	5.90	7.60	5.67
61.5	Isorhamnetin	2.51	3.07	5.58	4.59	7.55
62.0	Eupatolitin	2.28	2.39	3.82	3.83	3.32
63.5	Rosmarinic acid	6.73	7.45	8.10	4.39	5.64
63.6	β-santonin	7.39	8.98	8.41	8.08	6.01
63.8	Chrysosplenol D	7.16	9.94	7.91	8.31	8.20
64.1	Taurin ^3^	3.44	4.78	3.72	3.12	1.23
65.4	Cirsilineol	7.06	7.32	7.04	6.16	3.84
66.7	Casticin	8.58	4,87	8.81	8.32	8.23
68.7	Arteannuin b	4.05	–	–	–	–
72.2	Artemisinin ^2^	1.45	0.24	0.12	0.07	0.16

^1^ Retention time, min; ^2^ the identification of the peaks or the identities was confirmed by comparing them with authentic samples; ^3^ (3*S*,3a*S*,5a*R*,9b*S*)-3,5a,9-trimethyl-3a,5,5a,7,8,9b-hexahydronaphtho [1,2-b]furan-2,6(3*H*,4*H*)-dione.

**Table 3:** Antibacterial activity (MIC, MBC/MFC, µg/mL) of the ethanol extracts, namely *A. annua* cv. Novichok, *A. dracunculus* cv. Smaragd, *A. santonica* cv. Citral, *A. abrotanum* cv. Euxin, and *A. scoparia* cv. Tavrida.

Bacteria/Fungi Strain	*A. annua * cv. Novichok		*A. dracunculus* cv. Smaragd		*A. santonica* cv. Citral		*A. abrotanum* cv. Euxin		*A. scoparia* cv. Tavrida		Nor ^1^/Chl ^2^/Dif ^3^	
	MIC	MBC/MFC	MIC	MBC/MFC	MIC	MBC/MFC	MIC	MBC/MFC	MIC	MBC/MFC	MIC	MBC/MFC
*Rathayibacter iranicus* VKM Ac-162	500	1000	310	310	1000	1000	2000	2000	5000	5000	0.50	0.50
*Bacillus subtilis * VKM B-12	2000	>5000	2000	>5000	4000	>5000	4000	>5000	4000	>5000	0.50	0.50
*Agrobacterium tumefaciens * A-47	4000	4000	2000	2000	2000	2000	4000	4000	625	625	250.00	500.00
*Xanthomonas arboricola* S3	1000	2000	625	1250	560	1120	2000	4000	150	625	250.00	500.00
*Alternaria solani* K-100054	4000	4000	2500	2500	2500	2500	>5000	>5000	1250	1250	1.90	31.30
*Fusarium graminearum* FG-30	4000	>5000	2500	2500	2500	2500	>5000	>5000	310	625	3.90	62.50
*Rhizoctonia solani* VKM F-895	2000	2000	2500	5000	1120	1120	>5000	>5000	150	625	3.90	125.00

^1^ Norfloxacin; ^2^ chloramphenicol; ^3^ difenoconazole.

**Table 4:** Toxic effects of Artemisia extracts on *Caenorhabditis elegans* of the N2 wild-type line.

Sample	Percentage of Dead Nematodes, %					
	Extract Concentration, µg/mL					
	1000	500	250	125	62.5	31.25
*A. annua* cv. Novichok	100	92.0 ± 2.2	75.3 ± 3.5	55.3 ± 4.0	46.0 ± 4.0	22.0 ± 4.1
*A. dracunculus* cv. Smaragd	100	95.3 ± 1.7	68.0 ± 3.8	44.0 ± 4.0	34.0 ± 3.8	24.0 ± 4.2
*A. santonica* cv. Citral	100	94.5 ± 1.6	80.0 ± 2.8	67.0 ± 3.3	51.0 ± 3.5	40.0 ± 3.4

**Table 5:** Toxic effects of Artemisia extracts on *Caenorhabditis elegans*, both the N2 wild-type and mutant lines of RB896, RB756, JD217, and RB918.

Sample	Percentage of Dead Nematodes, %				
	N2	RB896 *gar-*1	RB756 *gar*-2	JD217 *gar*-3	RB918 *acr-*16
*A. annua* cv. Novichok	85.2 ± 2.2	9.5 ± 2.0	9.0 ± 2.0	83.6 ± 2.3	8.0 ± 1.9
*A. dracunculus* cv. Smaragd	82.4 ± 2.4	77.2 ± 2.6	72.0 ± 2.8	84.5 ± 2.5	83.0 ± 2.6
*A. santonica* cv. Citral	86.5 ± 2.4	48.0 ± 3.5	93.5 ± 1.7	27.5 ± 3.1	83.5 ± 2.6

Note: Concentration of extracts is 500 µg/mL.

**Table 6:** Toxic effects of Artemisia extracts on the *C. elegans* line of the N2 wild-type and the mutant lines of VC1041, VC 428, CB211, CB904, as well as CB522 VC1041, VC 428, CB211, CB904, and CB522.

Sample	Percentage of Dead Nematodes, %					
	N2	VC1041 *lev-*8	VC428 *unc-*63	CB211 *lev-*1	CB904 *unc-*38	CB522 *unc-*29
*A. annua* cv. Novichok	92.0 ± 1.7	85.5 ± 2.4	14.8 ± 2.2	46.5 ± 3.5	86.5 ± 2.4	90.8 ± 1.8
*A. dracunculus* cv. Smaragd	82.0 ± 2.7	55.2 ± 3.1	35.0 ± 3.3	28.5 ± 3.1	47.5 ± 3.5	67.6 ± 2.9
*A. santonica* cv. Citral	91.0 ± 2.0	67.5 ± 3.3	27.5 ± 3.1	66.0 ± 3.3	100	62.5 ± 3.4

Note: Concentration of extracts is 500 µg/mL.

## Data Availability

The data presented in this study are available upon request from the corresponding author.

## Supplementary Materials

The supporting information can be downloaded at: https://www.mdpi.com/article/10.3390/ijms241814372/s1.
