# Supplementary material for: Five Different Artemisia L. Species Ethanol Extracts’ Phytochemical Composition and Their Antimicrobial and Nematocide Activity
Source: Int J Mol Sci. 2023 Sep 21;24(18):14372. doi: 10.3390/ijms241814372 (PMC10532408; doi:10.3390/ijms241814372)
Supplement: Supplementary file 1 [file ijms-24-14372-s001.zip › ijms-2587775-supplementary.pdf]

Table S1. The results of determination of quantitative content of non-polar components in extracts of the plants as follows: *A. annua* cv. Novichok, A. dracunculus cv. Smaragd, A. santonica cv. Citral, A. abrotanum cv. Euxin, A. scoparia cv. Tavrida obtained by GC-MS (components with ω > 0.04%, mass., n = 3, p = 0.95)

**tR,[1] min**	**RI[2]**	**Compound**	**Molecular formula**	**m/z and peak relative intensity (%)[3]**	**ω (%)**				
					A. annua **cv. Novichok**	***A. dracunculus* cv. Smaragd**	***A. santonica* cv. Citral**	***A. abrotanum* cv. Euxin**	***A. scoparia* cv. Tavrida**
1	2	3	4	5	6	7	8	9	10
6.38	939	α-Pinene	$C_{10}H_{16}$	136([M+], 14%), 93(100 %), 91(47 %), 77(38 %)	0.523 ± 0.003	0.210 ± 0.002	7.940 ± 0.004	0.083 ± 0.005	5.240 ± 0.004
6.43	947	Camphene	$C_{10}H_{16}$	136([M+], 12 %), 121(38 %), 93(100 %), 79(77 %)	3.340 ± 0.005	1.104 ± 0.003	2.990 ± 0.002	8.104 ± 0.004	0.091 ± 0.002
6.66	978	β-Pinene	$C_{10}H_{16}$	136([M+], 16 %), 93(100 %), 91(31 %), 77(28 %)	0.841 ± 0.003	1.012 ± 0.005	0.540 ± 0.002	0.091 ± 0.004	5.684 ± 0.004
6.96	984	Sabinene	$C_{10}H_{16}$	136([M+], 18 %), 93(100 %), 91(38%), 77(79 %)	1.260 ± 0.003	0.062 ± 0.004	–	–	1.410 ± 0.002
7.08	988	β-Myrcene	$C_{10}H_{16}$	136([M+], 16 %), 93(86 %), 91(10 %), 69(80 %)	9.820 ± 0.002	1.350 ± 0.003	8.020 ± 0.002	0.064 ± 0.003	0.541 ± 0.005
7.11	1010	3-Carene	$C_{10}H_{16}$	136([M+], 15 %), 93(100 %), 91(50 %), 79(32 %)	2.160 ± 0.004	1.302 ± 0.003	0.093 ± 0.002	0.052 ± 0.003	0.043 ± 0.004
7.83	1024	Limonene	$C_{10}H_{16}$	136([M+], 23 %), 121(19 %), 93(60 %), 68(100 %)	1.872 ± 0.004	3.612 ± 0.003	3.542 ± 0.004	–	2.800 ± 0.004
7.90	1040	*cis*-β-Ocimene	$C_{10}H_{16}$	136([M+], 12 %), 93(100 %), 79(31 %), 41(36 %)	1.020 ± 0.002	1.370 ± 0.004	1.232 ± 0.003	–	–
7.78	1046	Zineol	$C_{10}H_{18}O$	154([M+], 36 %), 108(56 %), 81(65 %) 43(100 %),	11.760 ± 0.0100	4.231 ± 0.005	1.563 ± 0.004	–	0.113 ± 0.002
7.99	1050	*trans*-β-Ocimene	$C_{10}H_{16}$	136([M+], 14 %), 93(100 %), 79(30 %), 41(32 %)	1.022 ± 0.004	0.051 ± 0.003	14.513 ± 0.012	23.214 ± 0.050	0.086 ± 0.0021
8.17	1071	Artemisia ketone	$C_{10}H_{16}O$	153([M+H]+, 20 %), 83(100 %), 55(30 %), 39(71 %)	32.810 ± 0.050	–	1.212 ± 0.002	–	0.048 ± 0.004
8.53	1083	Artemisia alcohol	$C_{10}H_{18}O$	155([M+H]+, 15 %), 85(100 %), 41(31 %), 55 (13%)	3.744 ± 0.010	0.061 ± 0.003	0.053 ± 0.002	–	0.084 ± 0.004
8.94	1089	*cis*-sabinenehydrate	$C_{10}H_{18}O$	155([M+H]+, 25 %), 93 (94 %), 71 (80 %), 43(100 %)	–	–	1.583 ± 0.004	0.080 ± 0.003	–
9.71	1145	Camphor	$C_{10}H_{16}O$	152([M+], 25 %), 108(36 %), 95(100 %), 81(72 %)	10.102 ± 0.011	–	1.324 ± 0.002	1.290 ± 0.004	–
9.86	1164	*cis*-Chrysanthenol	$C_{10}H_{16}O$	152([M+], 24 %), 121(88 %), 109(100 %), 81(89 %)	–	–	21.570 ± 0.033	34.260 ± 0.050	–
10.07	1167	Borneol	$C_{10}H_{18}O$	154([M+], 14 %), 110(29 %), 95(100 %), 93(9 %)	1.953 ± 0.003	–	–	1.432 ± 0.004	2.053 ± 0.003
10.37	1178	Estragole	$C_{10}H_{12}O$	148([M+], 100 %), 147(60 %), 121(44 %), 77(44 %)	–	56.200 ± 0.060	4.860 ± 0.010	3.340 ± 0.005	–
10.90	1273	α-Citral	$C_{10}H_{16}O$	152([M+], 19 %), 84(26 %), 69(100 %), 41(92 %)	0.626 ± 0.004	1.280 ± 0.003	2.850 ± 0.004	0.350 ± 0.002	1.830 ± 0.010
11.30	1277	β-Citral	$C_{10}H_{16}O$	152([M+], 17 %), 69(85 %), 41(100 %), 27(26 %)	1.020 ± 0.003	0.913 ± 0.004	3.741 ± 0.004	0.383 ± 0.002	2.020 ± 0.005
12.53	1360	Eugenol	$C_{10}H_{12}O_2$	164([M+], 100 %), 149(36 %), 103(36 %), 77(34 %)	–	–	4.733 ± 0.020	–	1.700 ± 0.010
13.63	1419	Caryophyllene	$C_{15}H_{24}$	205([M+H]+, 72 %), 91(85 %), 93(100 %), 33(92 %),	0.732 ± 0.002	–	0.044 ± 0.003	0.052 ± 0.004	3.020 ± 0.003

13.79	1454	(*E*)-β-Famesene	$C_{15}H_{24}$	205([M+H]+, 25 %), 93(45 %), 69 (78 %), 41 (100 %)	1.963 ± 0.003	–	1.053 ± 0.002	0.193 ± 0.004	–
14.23	1479	α-Curcumene	$C_{15}H_{22}$	202([M+], 23 %), 132(75 %), 105(46 %), 119(100 %)	–	–	0.132 ± 0.005	–	2.340 ± 0.004
14.39	1482	Germacrene D	$C_{15}H_{24}$	204([M+], 22 %), 161(100 %), 105(76 %), 91(68 %)	–	–	0.070 ± 0.004	–	–
14.41	1485	β-Selinene	$C_{15}H_{24}$	204([M+], 88 %), 107(89%), 105(90%), 41(100%)	1.704 ± 0.003	–	3.294 ± 0.005	–	–
14.50	1502	Capillene	$C_{12}H_{10}$	153([M+], 100 %), 154(90%), 152(94%), 115(26%)	–	0.043 ± 0.004	1.234 ± 0.003	3.540 ± 0.002	44.610 ± 0.060
15.54	1572	Capillin	$C_{12}H_{8}O$	168([M+], 51 %), 140(100%), 139 (88%), 91(65%)	–	0.053 ± 0.005	0.871 ± 0.003	–	7.460 ± 0.030
16.07	1578	Spathulenol	$C_{15}H_{24}O$	220([M+], 24 %), 205(61%), 43(100%), 41(62%)	0.155 ± 0.005	2.890 ± 0.002	0.824 ± 0.003	–	3.830 ± 0.005
16.15	1604	Isospathulenol	$C_{15}H_{24}O$	220([M+], 15 %), 119(100%), 91(60%), 43 (65%)	–	1.601 ± 0.004	5.478 ± 0.002	0.062 ± 0.005	–
16.45	1656	α-Eudesmol	$C_{15}H_{26}O$	222([M+], 10%), 189(44%), 149(51%), 59(100%)	0.083 ± 0.004	–	0.172 ± 0.003	1.431 ± 0.005	–
16.63	1686	α-Bisabolol	$C_{15}H_{26}O$	223([M+H]+, 30 %), 69(80 %), 43(100 %), 41 (89%)	0.404 ± 0.003	–	–	8.183 ± 0.040	0.522 ± 0.005
17.12	1694	Arteannuin b	$C_{15}H_{20}O_{3}$	249([M+H]+, 28 %), 55(78%), 43(100%), 41(83%)	4.001 ± 0.010	–	–	–	–
18.22	1826	Arteannuic acid	$C_{15}H_{22}O_{2}$	234([M+], 33 %), 121(100%), 119(43%), 93(42%),	4.050 ± 0.020	1.240 ± 0.002	–	–	0.864 ± 0.003
19.09	1884	(2*E*,4*E*)-*N*-Isobutyl-2,4-undecadiene-8,10-diynamide	$C_{15}H_{19}NO$	229([M+], 27 %), 157(100%), 128(55%), 57(30%)	0.055± 0.004	2.610 ± 0.005	0.152 ± 0.004	0.540 ± 0.005	–
19.46	1943	Pellitorine	$C_{14}H_{25}NO$	223([M+], 25 %), 151(100%), 96(51%), 81(64%),	–	3.640 ± 0.010	1.973 ± 0.005	0.930 ± 0.003	–
19.52	1985	Scopoletin	$C_{10}H_{18}O_{4}$	192([M+], 100 %), 177(61%), 149(51%), 69(35%)	2.082 ± 0.010	–	–	–	–
20.58	2018	Scoparone	$C_{11}H_{10}O_{4}$	206([M+], 100%), 191(40%), 178(14%), 163(18%)	–	–	0.262 ± 0.003	2.010 ± 0.004	10.930 ± 0.030
20.60	2086	Isofraxidin	$C_{11}H_{10}O_{5}$	222([M+], 100%), 207(29%), 179(30%), 123(36%)	0.084 ± 0.002	0.243 ± 0.003	–	0.104 ± 0.003	–
20.75	2111	Phytol	$C_{20}H_{40}O$	300([M+H]+, 25 %), 123(27%), 71(100%), 68(23%)	0.814 ± 0.003	1.680 ± 0.003	–	10.010 ± 0.020	0.980 ± 0.004
21.02	2140	(2*E*,4*E*)-1-(piperidin-1-yl)deca-2,4-dien-1-one	$C_{15}H_{25}NO$	235([M+], 39 %), 192(82%), 84(100%), 81(72%)	–	3.190 ± 0.010	–	–	–
21.14	2635	7-Geranyloxycoumarin	$C_{19}H_{22}O_{3}$	299([M+H]+, 15 %), 163(43%), 162(58%), 69(100%)	–	10.052 ± 0.040	2.254 ± 0.010	0.202 ± 0.003	–
29.76	3065	α-Amyrin	$C_{30}H_{50}O$	426([M+], 21 %), 219(18%), 218(100%), 55(16%)	–	–	–	–	1.701 ± 0.005

[1] retention time, min. [2] retention index, [3] $[M^+]$ (EI at 70 eV) and $[M + H]^+$ (CI at 30 eV).

Table S2. The results of quantitative content determination for polar components in the extracts of *A. annua* cv. Novichok, *A. dracunculus* cv. Smaragd, *A. santonica* cv. Citral, *A. abrotanum* cv. Euxin, *A. scoparia* cv. Tavrida obtained by HPLC–MS/MS(ESI+) technique (the components with ω ≥ 0.04%, in mean ± SEM, *n* = 3, *p* = 0.95 are provided)

tR, [1] min	Compound	Molecular Formula	mode ESI+				ω (%)				
			Exp. Mass [2], m/z	Calc. Mass, m/z	Δ, ppm	MS/MS, m/z	*A. annua* cv. Novichok	*A. dracunculus* cv. Smaragd	*A. santonica* cv. Citral	*A. abrotanum* cv. Euxin	*A. scoparia* cv. Tavrida
1	2	3	4	5	6	7	8	9	10	11	12
2.4	Arteannuic acid	$C_{15}H_{22}O_2$	235.1699	235.1693	0.06	190.1703, 217.1572	4.850 ± 0.022	1.740 ± 0.032	–	–	0.921 ± 0.020
15.0	Gentisic acid	$C_7H_6O_4$	155.0345	155.0339	0.06	137.0233, 111.0439	0.572 ± 0.050	0,520 ± 0.030	0.482 ± 0.020	0.097± 0.020	0.580 ± 0.042
33.5	Scopolin	$C_{16}H_{18}O_9$	355.1032	355.1024	0.08	193.0502, 178.0250	2.089 ± 0.031	–	–	–	–
35.8	Isofraxidin	$C_{11}H_{10}O_5$	223.0614	223.0601	0.13	208.0366, 163.0390	1.110 ± 0.020	2.282 ± 0.030	2.543 ± 0.030	2.223 ± 0.020	2.121± 0.030
36.7	Feruloyl glucose	$C_{16}H_{20}O_9$	357.1184	357.1180	0.04	339.1082, 177.0563	1.712 ± 0.012	4.924 ± 0.010	1.534 ± 0.020	4.632 ± 0.020	4.231± 0.031–
38.5	Vicenin-2	$C_{27}H_{30}O_{15}$	595.1687	595.1657	0.30	577.1544, 457.1094	0.062 ± 0.004	4.608 ± 0.020	–	2.042 ± 0.005	6.084 ± 0.020
40.8	3-*O*-Feruloyl-quinic acid	$C_{17}H_{20}O_9$	369.1208	369.1180	0.30	177.0574, 145.0284	3.842 ± 0.020	4.610 ± 0.040	2.732 ± 0.050	4.521 ± 0.030	4.320 ± 0.020
46.8	Scopoletin [3]	$C_{10}H_8O_4$	193.0531	193.0495	0.30	178.0258, 165.0581	2.064 ± 0.011	–	–	–	–
49.9	Fraxidin	$C_{11}H_{10}O_5$	223.0607	223.0601	0.06	190.0262, 162.0312	1.040 ± 0.005	2.046 ± 0.005	2.052 ± 0.010	2.023 ± 0.010	1.608 ± 0.010
51.3	Rutin [3]	$C_{27}H_{30}O_{16}$	611.1648	611.1607	0.41	465.1074, 303.0508	3.231 ± 0.020	2.280 ± 0.020	4.141 ± 0.010	5.040 ± 0.030	2.983 ± 0.010
52.3	Isoquercetin	$C_{21}H_{20}O_{12}$	465.1043	465.1028	0.15	303.0491, 145.0531	3.390 ± 0.030	3.913 ± 0.020	4.251 ± 0.040	3.350 ± 0.012	0.630 ± 0.010
53.5	Quercetin-3-*O*-hexoside	$C_{21}H_{20}O_{12}$	465.1038	465.1028	0.10	447.0934, 303.0499	4.735 ± 0.030	2.940 ± 0.030	4.831 ± 0.020	4.832 ± 0.020	5.332 ± 0.030
53.8	Luteolin 7-*O*-glucoside	$C_{21}H_{20}O_{11}$	449.1090	449.1078	0.30	287.0564, 153.0191	3.434 ± 0.050	3.323 ± 0.040	3.341 ± 0.010	2.785 ± 0.030	7.242 ± 0.010
57.2	Isochlorogenic acid	$C_{25}H_{24}O_{12}$	517.1348	517.1341	0.07	499.1260, 163.0394	3.043 ± 0.005	3.450 ± 0.020	3.561 ± 0.011	3.216 ± 0.020	6.343 ± 0.020
58.5	Isorhamnetin-3-rutinoside	$C_{28}H_{32}O_{16}$	625.1771	625.1763	0.08	479.1243, 317.0691	4.437 ± 0.030	3.345 ± 0.050	3.231 ± 0.030	4.221 ± 0.020	–
58.9	Quercetrin	$C_{21}H_{20}O_{11}$	449.1089	449.1078	0.11	303.0473, 147.0748	6.405 ± 0.010	6.740 ± 0.020	7.690 ± 0.040	6.531 ± 0.010	2.100 ± 0.050
61.4	Luteolin [3]	$C_{15}H_{10}O_6$	287.0563	287.0550	0.13	153.0180, 135.0433	3.320 ± 0.020	3.570 ± 0.010	5.902 ± 0.050	7.603 ± 0.020	5.671 ± 0.010
61.5	Isorhamnetin	$C_{16}H_{12}O_7$	317.0662	317.0656	0.06	302.0434, 153.0194	2.514 ± 0.050	3.070 ± 0.020	5.581 ± 0.020	4.591 ± 0.040	7.550 ± 0.030
62.0	Eupatolitin	$C_{17}H_{14}O_8$	347.0774	347.0761	0.13	332.0526, 197.0443	2.280 ± 0.020	2.394 ± 0.030	3.823 ± 0.040	3.834 ± 0.030	3.321 ± 0.020
63.5	Rosmarinic acid	$C_{18}H_{16}O_8$	361.0926	361.0918	0.08	163.0398, 181.0500	6.730 ± 0.010	7.450 ± 0.030	8.102 ± 0.010	4.391 ± 0.020	5.644 ± 0.040
63.6	β-Santonin	$C_{15}H_{18}O_3$	247.1337	247.1329	0.08	229.1219, 173.0962	7.390 ± 0.020	8.980 ± 0.030	8.407 ± 0.020	8.082 ± 0.040	6.014 ± 0.050
63.8	Chrysosplenol D	$C_{18}H_{16}O_8$	361.0933	361.0918	0.15	346.0675, 328.0562	7.167 ± 0.030	9.940 ± 0.010	7.911 ± 0.020	8.312 ± 0.010	8.203 ± 0.010
64.1	Taurin [4]	$C_{15}H_{20}O_3$	249.1493	249.1485	0.08	176.1201, 160.0882	3.440 ± 0.020	4.782 ± 0.012	3.723 ± 0.011	3.123 ± 0.020	1.232 ± 0.030
65.4	Cirsilineol	$C_{18}H_{16}O_7$	345.0983	345.0969	0.14	312.0612, 284.0662	7.060 ± 0.030	7.321 ± 0.040	7.036± 0.050	6.157± 0.030	3.844± 0.020
66.7	Casticin	$C_{19}H_{18}O_8$	375.1079	375.1074	0.05	360.0851, 342.0743	8.583 ± 0.040	4,871 ± 0.020	8.805 ± 0.050	8.321± 0.040–	8.232 ± 0.020
68.7	Arteannuin b	$C_{15}H_{20}O_3$	249.1493	249.1483	0.10	231.1385, 185.0967	4.051 ± 0.030	–	–	–	–
72.2	Artemisinin [3]	$C_{15}H_{22}O_5$	283.1582	283.1540	0.42	265.1426, 247.1321	1.451 ± 0.050	0.244 ± 0.040	0.121 ± 0.020	0.073 ± 0.020	0.163± 0.030

[1] retention time, min; [2] Precursor Type $[M+H]^+$; [3] the identification of the peaks or the identities were confirmed by comparing them with authentic samples; [4] (3*S*,3a*S*,5a*R*,9b*S*)-3,5a,9-trimethyl-3a,5,5a,7,8,9b-hexahydronaphtho [1,2-b]furan-2,6(3*H*,4*H*)-dione.

Table S3. Antibacterial activity (MIC, MBC/MFC, µg/mL) of the ethanol extracts, namely *A. annua* cv. Novichok, *A. dracunculus* cv. Smaragd, *A. santonica* cv. Citral, *A. abrotanum* cv. Euxin, *A. scoparia* cv. Tavrida.

Bacteria/Fungi Strain	*A. annua* cv. Novichok		*A. dracunculus* cv. Smaragd		*A. santonica* cv. Citral		*A. abrotanum* cv. Euxin		*A. scoparia* cv. Tavrida		Nor [1]/Chl [2]/ Dif [3]	
	MIC	MBC/MFC	MIC	MBC/MFC	MIC	MBC/MFC	MIC	MBC/MFC	MIC	MBC/MFC	MIC	MBC/MFC
Rathayibacter iranicus VKM Ac-162	500±25	1000±50	310±30	310±30	1000±50	1000±50	2000±100	2000±100	5000±250	5000±250	0.50±0.03	0.50±0.03
Bacillus subtilis VKM B-12	2000±130	>5000	2000±100	>5000	4000±200	>5000	4000±200	>5000	4000±200	>5000	0.50±0.03	0.50±0.03
Agrobacterium tumefaciens A-47	4000±300	4000±300	2000±150	2000±150	2000±150	2000±150	4000±300	4000±300	625±40	625±40	250.00±22.5	500.00±42.20
Xanthomonas arboricola S3	1000±50	2000±100	625±40	1250±56	560±28	1120±56	2000±150	4000±300	150±10	625±40	250.00±23.10	500.00±35.60
Alternaria solani K-100054	4000±400	4000±400	2500±250	2500±250	2500±250	2500±250	>5000	>5000	1250±125	1250±125	1.90±0.20	31.30±3.10
Fusarium graminearum FG-30	4000±200	>5000	2500±125	2500±125	2500±125	2500±125	>5000	>5000	310±20	625±40	3.90±0,40	62.50±6.00
Rhizoctonia solani VKM F-895	2000±100	2000±100	2500±125	5000±250	1120±56	1120±56	>5000	>5000	150±10	625±40	3.90±0.22	125.00±11.10

[1] Norfloxacin; [2] chloramphenicol; [3] difenoconazole
